# Chloroplast pH Homeostasis for the Regulation of Photosynthesis

**DOI:** 10.3389/fpls.2022.919896

**Published:** 2022-05-25

**Authors:** Mai Duy Luu Trinh, Shinji Masuda

**Affiliations:** ^1^Department of Plant and Environmental Sciences, University of Copenhagen, Copenhagen, Denmark; ^2^Department of Life Science and Technology, Tokyo Institute of Technology, Yokohama, Japan

**Keywords:** chloroplast, pH homeostasis, non-photochemical quenching, photosynthesis, ΔpH

## Abstract

The pH of various chloroplast compartments, such as the thylakoid lumen and stroma, is light-dependent. Light illumination induces electron transfer in the photosynthetic apparatus, coupled with proton translocation across the thylakoid membranes, resulting in acidification and alkalization of the thylakoid lumen and stroma, respectively. Luminal acidification is crucial for inducing regulatory mechanisms that protect photosystems against photodamage caused by the overproduction of reactive oxygen species (ROS). Stromal alkalization activates enzymes involved in the Calvin–Benson–Bassham (CBB) cycle. Moreover, proton translocation across the thylakoid membranes generates a proton gradient (ΔpH) and an electric potential (ΔΨ), both of which comprise the proton motive force (*pmf*) that drives ATP synthase. Then, the synthesized ATP is consumed in the CBB cycle and other chloroplast metabolic pathways. In the dark, the pH of both the chloroplast stroma and thylakoid lumen becomes neutral. Despite extensive studies of the above-mentioned processes, the molecular mechanisms of how chloroplast pH can be maintained at proper levels during the light phase for efficient activation of photosynthesis and other metabolic pathways and return to neutral levels during the dark phase remain largely unclear, especially in terms of the precise control of stromal pH. The transient increase and decrease in chloroplast pH upon dark-to-light and light-to-dark transitions have been considered as signals for controlling other biological processes in plant cells. Forward and reverse genetic screening approaches recently identified new plastid proteins involved in controlling ΔpH and ΔΨ across the thylakoid membranes and chloroplast proton/ion homeostasis. These proteins have been conserved during the evolution of oxygenic phototrophs and include putative photosynthetic protein complexes, proton transporters, and/or their regulators. Herein, we summarize the recently identified protein players that control chloroplast pH and influence photosynthetic efficiency in plants.

## Introduction: Plastidial pH as a Signal Regulating Chloroplast Activity

To adapt to environmental fluctuations, plants demonstrate the developed ability to monitor abiotic and biotic parameters using sensors and receptors in different subcellular compartments, which then induces signal transduction networks for activating adaptive responses (Baier et al., 2008). As an important cellular compartment, chloroplasts are dynamic and specific sensors of intra- and extra-cellular stimuli such as light and CO_2_ (Bobik and Burch-Smith, 2015). The ability to sense light intensity and CO_2_ concentration is essential for chloroplasts and the photosynthetic apparatus to perform photosynthesis effectively and to fine-tune the mechanisms that protect against unfavorable conditions.

Busa and Nuccitelli first proposed the importance of changes in intracellular pH for metabolic regulation in a variety of animal cells (Busa and Nuccitelli, 1984). Since then, pH has also been considered as an important signal/messenger in plant cells (Felle, 2001). The signal for an ongoing process, while a messenger brings certain information, leads to a change of state in plants. For example, light-driven photosynthesis induces the acidification of thylakoid lumen, which activates photoprotective mechanisms such as non-photochemical quenching (NPQ). In other words, luminal pH is a signal that reflects changes in light intensity to control protective mechanisms against photodamage. Several luminal pH sensors have been reported to date; for example, PsbS (Krishnan-Schmieden et al., 2021), ATP synthase (Schwarz and Strotmann, 1998; Hahn et al., 2018), violaxanthin de-epoxidase (Hieber et al., 2000; Emanuelsson et al., 2003; Arnoux et al., 2009; Saga et al., 2010; Schaller et al., 2010), and plastocyanin (Sas et al., 2006) in land plants, light-harvesting complex stress-related protein3 (LHCSR3) in green algae (Ballottari et al., 2016), and photosystem I (PSI)-fucoxanthin-chlorophyll *a/c* protein complex in diatoms (Nagao et al., 2019). In the stroma, light-dependent alkalization activates fructose biphosphatase and ribulose-1,5-bisphosphate (RuBP) carboxylase, which are involved in the Calvin–Benson–Bassham (CBB) cycle (Lorimer et al., 1976; Flügge et al., 1980; Mott and Berry, 1986), as well as Triose Phosphate/phosphate Translocator1 (TPT1) and Phosphate Transporter2 (PHT2), which are involved in the import of inorganic phosphate (P_i_) from the cytosol to the chloroplast stroma (Flügge and Heldt, 1984; Versaw and Harrison, 2002). By contrast, a decrease in alkalization level in the stroma downregulates CO_2_ fixation (Demmig and Gimmler, 1979; Huber and Maury, 1980; Maury et al., 1981). Hence, the pH of stroma and thylakoid lumen is considered to function as a signal/messenger for various chloroplast-specific biological processes, which must be regulated precisely. Here, we summarize our current understanding of how chloroplast pH serves as an important messenger/signal for controlling chloroplast metabolism, and we discuss the potential mechanisms involved in regulation of chloroplast pH.

## Dynamics of Chloroplast pH

The importance of chloroplast pH homeostasis was first proposed in the 1990s, when alkalization of the stroma during light phase was shown to be essential for efficient assimilation of CO_2_ in the CBB cycle (Heldt et al., 1973; Werdan et al., 1975; Wagner et al., 1990; Wu and Berkowitz, 1992; Hauser et al., 1995). The pH of chloroplast stroma and thylakoid lumen is potentially influenced by proton-coupled electron transfer during photosynthesis as well as by stromal and luminal H^+^ buffering and changes in metabolic reactions (Buchanan, 1980, 2017; Maury et al., 1981; Peters and Berkowitz, 1991). Upon exposure to light, the pH of the stroma increases, whereas that of the thylakoid lumen decreases. The luminal pH has been estimated at 5.8–6.5 under normal light conditions and 4.5–4.8 under high light conditions (Kramer et al., 1999). Takizawa et al. reported a luminal pH of 7.5 under weak light and ambient CO_2_ conditions and 5.7 under saturating light and 50 ppm CO_2_ (Takizawa et al., 2007). Using pH-sensitive spin probes for electron paramagnetic resonance (EPR) measurement, luminal pH was estimated at ~5.4–5.7 in the state of photosynthetic control and ~5.7–6.0 under photophosphorylation conditions (Tikhonov et al., 2008). Conversely, stromal pH in the dark was reported to be ~7, which increased to ~7.8–8.0 in the light (Werdan and Heldt, 1972; Heldt et al., 1973; Werdan et al., 1975; Demmig and Gimmler, 1983; Robinson, 1985; Wu and Berkowitz, 1992). Recently, owing to the use of a pH indicator called BCECF-AM (2′,7′-bis(2-carboxyethyl)-5-(and-6)-carboxyfluorescein, acetoxymethyl ester), the stromal pH was reported to increase from 7.32 ± 0.02 in the dark to 7.55 ± 0.09 in the light within less than 1 min upon illumination (Su and Lai, 2017; Aranda Sicilia et al., 2021). The proton concentration gradient (ΔpH) across thylakoid membranes under steady light was reported to be ~1.8–2.1 (Tikhonov et al., 2008), indicating that the difference in pH between the stroma and thylakoid lumen upon exposure to light, reported previously, was reliable.

## NPQ as an Indicator of the Acidification of Thylakoid Lumen

Light energy absorbed by photosynthetic pigments is utilized for (i) photochemistry, in which the excited energy is used for charge separation within PSII, (ii) fluorescence emission (0.6%–3% of the absorbed photons), (iii) triplet excited chlorophyll (^3^Chl^*^) generation (4%–25% of the absorbed photons), which is stable and potentially reacts with O_2_ to produce ^1^O_2_^*^ [reactive oxygen species (ROS)], and (iv) thermal dissipation (qN) or NPQ to its surroundings (Müller et al., 2001). Plants maintain a low yield of steady-state fluorescence emission and ^3^Chl^*^ generation by controlling photochemical quenching (qP) and NPQ. Thus, NPQ is essential for quenching excited Chl^*^, thereby avoiding ^3^Chl^*^ accumulation and ROS generation under excessive light conditions. This mechanism is considered to be the fastest and most effective photoprotective mechanism in land plants, as it eliminates >75% of the excess light energy (Niyogi, 1999).

**Figure 1:**
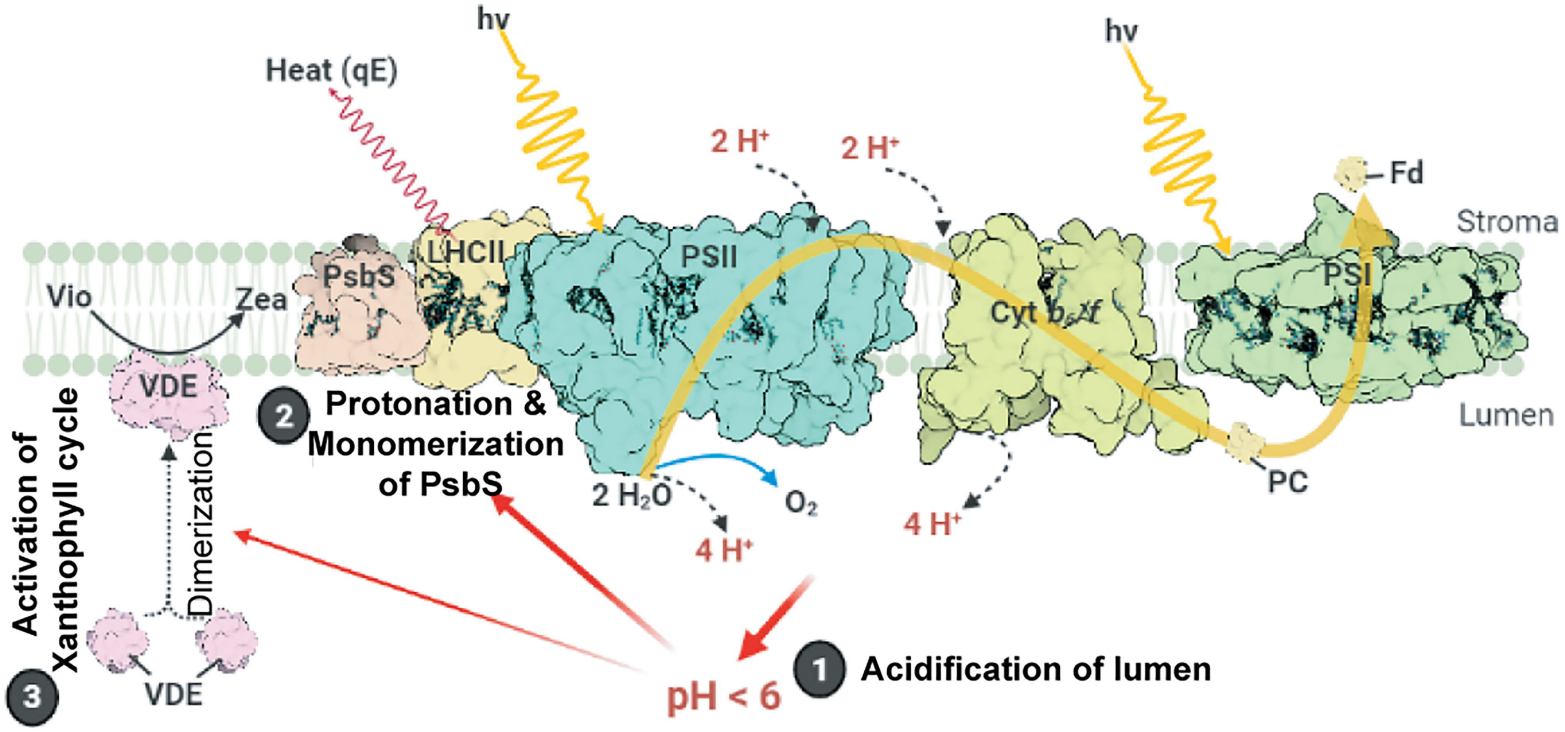
Schematic model of qE induction in higher plants. Photosystem II (PSII), cytochrome *b6f* (Cyt*b_6_f*), photosystem I (PSI), light-harvesting antenna complex II (LHCII), plastocyanin (PC), and ferredoxin (Fd) are shown. Light-induced ∆pH formation (lumen acidification) results from the oxidation of water at the oxygen evolving complex (OEC) in PSII and proton (H^+^) translocation during the Q cycle in Cyt*b_6_f*. Low luminal pH induces the protonation and monomerization of PsbS and activates violaxanthin de-epoxidase to convert violaxanthin (Vio) to zeaxanthin (Zea). Also, low luminal pH induces the aggregation of LHCII (for details, see text). These LHC conformational changes finally lead to the thermal dissipation of excess energy *via* the LHCs. This figure was designed using the BioRender web server (www.biorender.com).

The major NPQ component, energy-dependent quenching (qE), can be induced within a few seconds (Ruban, 2016) and relaxed within 1–2 min (Nilkens et al., 2010). The induction of qE relies on the (i) formation of ∆pH across thylakoid membranes, (ii) conversion of the xanthophyll cycle carotenoid violaxanthin to zeaxanthin, and (iii) protonation of the PSII protein subunit S (PsbS; Figure 1). Upon light illumination, the thylakoid lumen is acidified because of water oxidation at the oxygen-evolving complex (OEC) and proton translocation from the stroma to the thylakoid lumen. The lowered pH of the lumen activates the lipocalin family protein, violaxanthin de-epoxidase, which catalyzes the conversion of violaxanthin to zeaxanthin. The lowered luminal pH also induces the protonation of the carboxylate side chains of dimeric PsbS (Li et al., 2004), which in turn alters the interaction between PsbS and light-harvesting complex II (LHCII; Correa-Galvis et al., 2016; Dall’Osto et al., 2017; Sacharz et al., 2017), resulting in the induction of qE (Chmeliov et al., 2016; Nicol et al., 2019). A study on *Arabidopsis NoM* mutants, lacking all monomeric Lhcbs yet retaining full LHCII trimers, showed that the fast and slow activated qE are catalyzed within monomeric LHCs and LHCII trimers, respectively (Dall’Osto et al., 2017). Moreover, normal qE induction in WT is significantly reduced up to 60% in *Arabidopsis NoLHCII* mutants, lacking LHCII trimers, further supporting LHCII as the main quencher site (Nicol et al., 2019). The aggregation of both LHCIIs and minor LHC proteins (e.g., CP29 and CP26) is essential for qE induction (Ruban et al., 1992; Wentworth et al., 2001; Chmeliov et al., 2016, 2019; Farooq et al., 2018; van Amerongen and Chmeliov, 2020). This aggregation of LHCII is accelerated by the presence of zeaxanthin and high H^+^ concentration (Phillip et al., 1996; Ruban et al., 1997; Schaller et al., 2014).

## Light-Induced Formation of ΔpH Across Chloroplast Membranes

**Figure 2:**
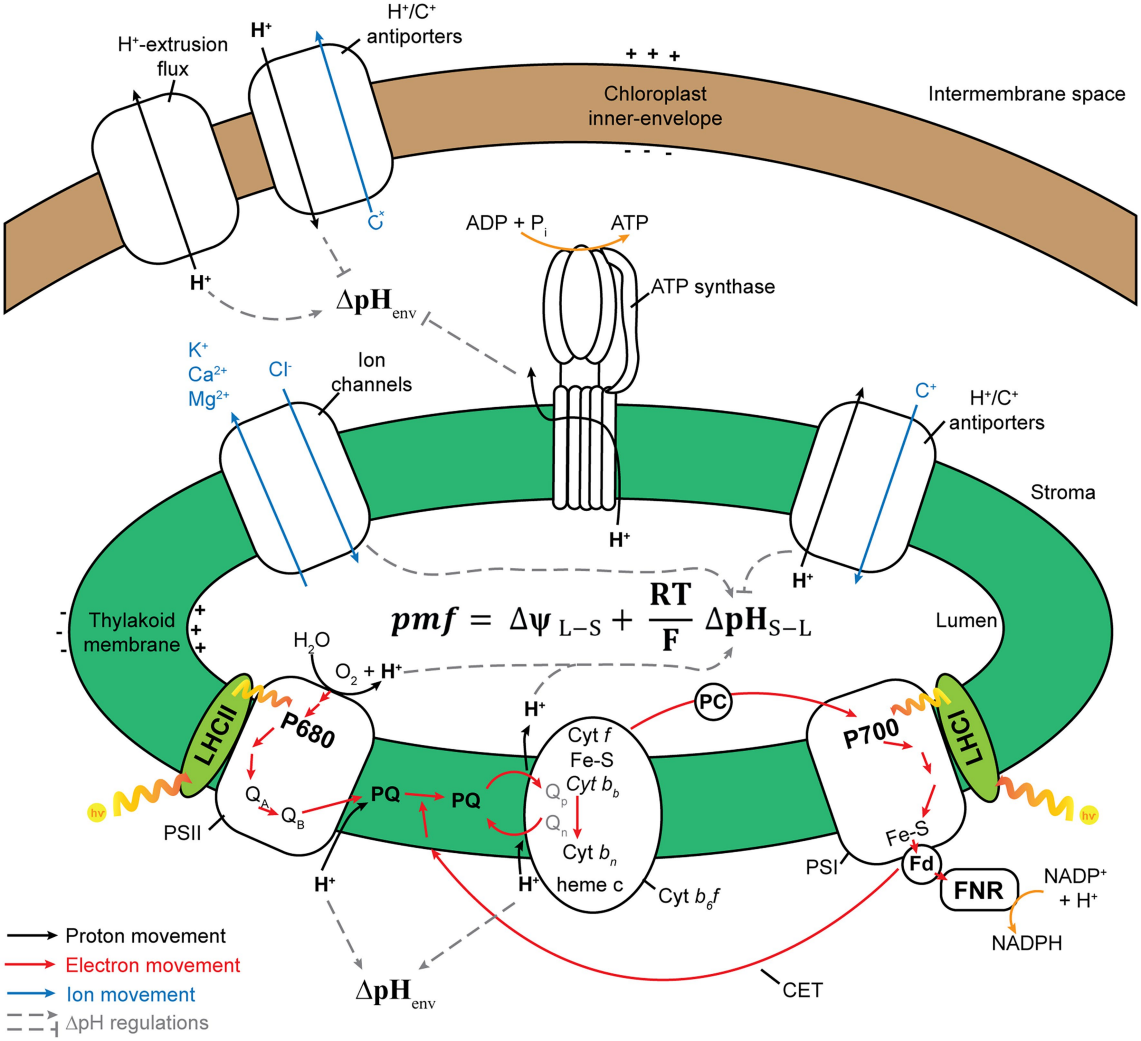
Schematic model of processes that contribute to proton motive force (*pmf*). PSII, photosystem II; Cyt*b_6_f*, cytochrome *b_6_f*; PQ, plastoquinone/plastoquinol pool; PSI, photosystem I; LHC, light-harvesting antenna complex; PC, plastocyanin; Fd, ferredoxin; CET, cyclic electron transfer; FNR, ferredoxin–NADPH reductase. Photosynthetic electron transfer reactions at the PSII and Cyt*b_6_f* complex are coupled to H^+^ transport from the stroma into the thylakoid lumen. These H^+^ are deposited in the lumen during the oxidation of water at the PSII and then transported across the membrane through PQ. The active transport of H^+^/ions from one side of the thylakoid membrane to another generates *pmf*. Alternative electron transport pathways through the CET modulate the ratio between the number of H^+^ translocated across the thylakoid membrane and the number of electrons transferred from water to NADPH. The chloroplast ATP synthase uses *pmf* and H^+^ efflux from the thylakoid lumen, along with the phosphorylation of ADP, to form ATP. By increasing or decreasing the ∆pH, ion flux *via* the thylakoid ion channels and H^+^ antiporters modulates the composition of *pmf*. The *pmf* (mV) is calculated using the following equation: *pmf* = ∆ψ_*L* − *S*_ + (RT/F)∆pH_*S* − *L*_, where ∆ψ_*L* − *S*_ denotes the electric potential between the lumen side and the stromal side of the thylakoid membrane; ∆pH_*S* − *L*_ denotes the H^+^ gradient between the stromal side and the lumen side of the thylakoid membrane; F denotes the Faraday constant; R denotes the gas constant; and T denotes the temperature. The H^+^ gradient across the chloroplast envelope membrane (∆pH_env_) is calculated using the following equation: ∆pH_env_ = pH_S_ – pH_C_, where pH_S_ denotes the stromal pH, and pH_C_ denotes the cytosol pH. This figure is adapted from Armbruster et al. (2017), Ptushenko et al. (2019), and Stirbet et al. (2019).

The concentration of proton (H^+^) in a cellular compartment determines its local pH; pH equals to −log_10_ [H^+^], where [H^+^] is proton concentration. The difference in pH between the stroma and the thylakoid lumen creates a transmembrane pH gradient or proton potential (ΔpH). Because H^+^ is charged, the difference in H^+^ concentration between the stroma and the thylakoid lumen also generates a trans-thylakoid electric field or electric potential (ΔΨ). The difference in H^+^ concentration across the thylakoid membranes establishes a H^+^ electrochemical potential difference or proton motive force (*pmf*), which is utilized to drive chloroplast ATP synthase for ATP synthesis (Baker et al., 2007). Light-induced photosynthetic electron transfer, coupled with proton translocation, generates ΔΨ and ΔpH across thylakoid membranes (Wilson et al., 2021). The formation of ΔpH and reduction in luminal pH are essential for photosynthetic regulatory mechanisms including the activation of NPQ (Schaller et al., 2014; Chmeliov et al., 2016, 2019; Ruban, 2016) and photosynthetic control of cytochrome *b_6_f* (Cyt*b_6_f*) activity (Hope et al., 1994; Kramer et al., 2003). Moreover, ΔpH and ΔΨ are thermodynamically equivalent components of *pmf* (Figure 2), following Mitchell’s chemiosmotic theory (Mitchell, 1961, 2011; Hangarter and Good, 1982; Wilson et al., 2021), which is indicated in the following equation:


$$pmf=\Delta \Psi_{L-S}-\frac{2.3\mathrm{RT}}{F}\times \Delta pH_{S-L}$$

where ΔΨ*_L−S_* is the electrical gradient across the thylakoid membrane (lumen–stroma), R is the gas constant, F is the Faraday constant, *T* is temperature, and ΔpH*_S−L_* is the proton gradient across the thylakoid membrane (pH_stroma_ − pH_lumen_; Armbruster et al., 2017; Dall’Osto et al., 2017; Wilson et al., 2021).

The *pmf* is consumed by thylakoid-localized ATP synthase for catalyzing the phosphorylation of ADP to produce ATP (Junge, 2004; Armbruster et al., 2017). The regulation of *pmf* formation and its composition (∆pH and ΔΨ) is necessary for photosynthetic regulation under fluctuating light conditions (Armbruster et al., 2017). The relative contributions of ∆pH and ΔΨ toward *pmf* are modulated by ion transport systems in the thylakoid membrane (Armbruster et al., 2016; Herdean et al., 2016; Wang et al., 2017). Under high light conditions, *pgr5* and *hope2/cfq* mutants, which lack PGR5/PGRL1-dependent cyclic electron transfer (CET), named after proton gradient regulation (PGR) 5 and PGR5-like1 (PGRL1) proteins (Munekage et al., 2002; DalCorso et al., 2008), and enhance the H^+^ efflux activity of chloroplast ATP synthase (Kanazawa et al., 2017; Takagi et al., 2017), respectively, exhibit lowered *pmf* levels and higher PSI photoinhibition than the wild type (WT). Contrarily, *minira*/*cgl160* and *vccn1*/*best1* mutants, which possess lower levels of plastidial ATP synthase in comparison with those in the WT (Fristedt et al., 2015; Davis et al., 2016) and are devoid of the Cl^−^ channel VCCN1/BEST1 (Duan et al., 2016; Herdean et al., 2016), respectively, exhibit elevated *pmf* levels and PSII photoinhibition. Lowered *pmf* in *pgr5* is mainly caused by lowered ∆pH formation, whereas elevated *pmf* in *miniral/cgl160* and *vccn1/best1* is mainly caused by ΔΨ increment, which demonstrates that the dissipation of ΔΨ is essential for avoiding PSII photoinhibition, while a high capacity for ∆pH helps plants in protecting PSI against photoinhibition. In other words, the precise regulation of *pmf* composition is essential for photoprotection (Armbruster et al., 2017).

The proton gradient across envelope membranes, ΔpH_env_ (Figure 2), caused by the difference in pH between chloroplast stroma (alkaline pH) and cytosol (nearly neutral pH, ~7.1−7.5), may contribute to the transport of ions and metabolites across the chloroplast envelope through proton-based exchangers, antiporters, and symporters. For example, Na^+^ and K^+^ are transported into chloroplasts by Na^+^/H^+^ and K^+^/H^+^ antiporters such as NHD1, thylakoid K^+^ efflux antiporter1 (KEA1), and KEA2 (Kunz et al., 2014), leading to the establishment of Na^+^ and K^+^ gradients across the envelope. These ion gradients can then be used to transport essential metabolites into chloroplasts, as in the case of BASS2, a Na^+^-dependent pyruvate transporter (Furumoto et al., 2011). However, the possibility exists that these ion/proton antiporters perform little function under light or are inhibited upon illumination, since photosynthesis induces cellular alkalization in the chloroplast stroma, mitochondrial matrix, and cytosol (Elsässer et al., 2020). Indeed, alkalization of the cytosol in mesophyll cells upon illumination was reported previously; however, the induced alkaline pH in the cytosol was transient, and CO_2_ inhibits the cytosolic alkalization in both C4 and C3 plants (Yin et al., 1990; Raghavendra et al., 1993). Elsässer et al. (2020) monitored pH changes in the chloroplast stoma, mitochondrial matrix, and the cytosol in mesophyll cells upon illumination and strikingly showed that cytosolic pH was maintained at alkaline levels during illumination periods (Elsässer et al., 2020). Although the study by Elsässer et al. (2020) has not yet been peer-reviewed, this work will challenge our previous knowledge of the tightly regulated homeostasis of cytosolic pH (Felle, 2001; Pittman, 2012; Sze and Chanroj, 2018; Wegner and Shabala, 2020; Zhou et al., 2021).

## H^+^-Dependent Transporters and Regulators Controlling Luminal pH

It has been well established that ΔpH across thylakoid membranes is mainly generated by H^+^ translocation from the stroma to the lumen through Cyt*b_6_f* activity (Malone et al., 2021), and it is relieved upon H^+^ flux from the lumen to the stroma *via* chloroplast ATP synthase (Hahn et al., 2018). Table 1 summarizes additional transporters and regulators controlling ΔpH as well as luminal pH.

**Table 1:** Proton-involved transporters and protein factors regulate thylakoid luminal pH.

Locus	Protein	Subcellular localization	Proposed function(s)	References
At4g04850	KEA3	Thylakoid membranes	K^+^/H^+^ antiporter NPQ control pH homeostasis	Chanroj et al., 2012 Kunz et al., 2014 Armbruster et al., 2016 Wang et al., 2017 Wang and Shikanai, 2019
At1g64150	CCHA1 PAM71 BICAT1	Thylakoid membranes	Putative Ca^2+/^H^+^ antiporter Mn^2+^, Ca^2+^, and pH homeostasis in chloroplasts Bivalent cation transporter	Wang et al., 2016 Schneider et al., 2016 Frank et al., 2019
At2g05620	PGR5	Thylakoid membranes	Proton-gradient regulation Main component/regulator of the PGR5-dependent cyclic electron flow Regulation of the linear electron flow Regulation of Q cycles in Cyt b_6_f*	Munekage et al., 2002 Nandha et al., 2007 DalCorso et al., 2008 Takagi and Miyake, 2018 Buchert et al., 2020 Wu et al., 2021
At4g31390	PGR6 ABC1K1 kinase	Chloroplast plastoglobules	An ABC1 (activity of Cytochrome bc1) atypical kinase phosphorylates VTE1 in tocopherol metabolism Plastoquinone homeostasis	Martinis et al., 2014 Pralon et al., 2019 Ksas et al., 2022
At1g54520	FLAP1	Chloroplast envelope and thylakoid membranes	NPQ control under fluctuating light conditions Regulation of luminal acidification Proton-gradient regulation	Sato et al., 2017 Trinh et al., 2019

* A study on *Chlamydomonas reinhardtii.*

### Ion/H^+^ Transport Through KEA3 and CCHA1/PAM71/BICAT1

Studies on KEA3 suggest that the modulation of *pmf* composition is also important for efficient photosynthesis. Like KEA1 and KEA2, KEA3 also belongs to the monovalent cation/H^+^ antiporter (CPA) superfamily (Chanroj et al., 2012). Although KEA3 is recognized as a K^+^/H^+^ antiporter (Chanroj et al., 2012), only its K^+^ transport activity could be confirmed through a complementation assay using the *Escherichia coli* K^+^ uptake-deficient mutant (Tsujii et al., 2019). The H^+^ transport activity of KEA3 has not yet been demonstrated. KEA3 contains an extended N-terminus, 13 transmembrane helixes, and a C-terminal regulatory nucleotide-binding KTN domain. The topology of the KTN domain is unclear but has been proposed to localize in the lumen (Armbruster et al., 2016) or in the stroma (Wang et al., 2017). The KTN domain downregulates KEA3 activity under high light conditions (Armbruster et al., 2016), possibly through interactions between the KTN domain and chloroplast nucleotides, such as NADPH/NADP^+^ and ATP/ADP. In previous studies, the Arabidopsis *kea3* loss-of-function mutant exhibited slower qE relaxation than the WT when light intensity shifted from high to low (Armbruster et al., 2014, 2016; Wang et al., 2017). Under these conditions, the ΔpH of *kea3* increased, whereas its *pmf* was affected and remained the same as that of the WT. These observations indicate that KEA3 alters the *pmf* composition to obtain high ΔΨ by transporting H^+^ and K^+^ across the thylakoid membranes during qE relaxation (Armbruster et al., 2017). The *kea3* mutant exhibited retarded growth compared with the WT under fluctuating light conditions (Armbruster et al., 2016), indicating that KEA3 activity is important for the rapid adjustment of LHCII from the energy (heat) dissipation mode under high light to the energy absorption mode under low light (Demmig-Adams et al., 2012).

In addition to KEA3, a putative thylakoid membrane-localized Mn^2+^ or Ca^2+^/H^+^ antiporter, named as CCHA1 (Wang et al., 2016), PAM71 (Schneider et al., 2016), or BICAT1 (Frank et al., 2019), may contribute to the modulation of *pmf* composition. CCHA1 localizes in the thylakoid membrane (Schneider et al., 2016; Frank et al., 2019). Topological analysis of CCHA1 suggests that its C-terminus is exposed to the luminal side of thylakoid membranes (Schneider et al., 2016). CCHA1 binds to divalent cations, as it contains two highly conserved E-x-G-D-(KR)-(TS) motifs (Schneider et al., 2016; Wang et al., 2016; Frank et al., 2019). Contradicting reports exist on the function of CCHA1 in chloroplast Ca^2+^ homeostasis regulation. Knockout of *CCHA1* induced the accumulation of Ca^2+^ in the thylakoid lumen (Schneider et al., 2016) and cytosol (Wang et al., 2016) and that of Mn^2+^ in the stroma (Schneider et al., 2016). By contrast, the *bicat1-1* mutant exhibited significantly lower Ca^2+^ uptake by thylakoids compared with the WT (Frank et al., 2019). Consistently, *ccha1*, *pam71*, and *bicat1-1* mutants displayed decreased NPQ induction under steady-state illumination conditions than the WT (Schneider et al., 2016; Wang et al., 2016; Frank et al., 2019). Unlike the WT, *pam71* mutant exhibited higher *pmf* values, with enhanced ΔΨ and reduced ΔpH (Schneider et al., 2016). Additionally, *pam71* and *ccha1* mutants exhibited lower proton conductivity (g_H_^+^) and chloroplast H^+^-ATPase activity, respectively, than the WT (Schneider et al., 2016; Wang et al., 2016). These results support the hypothesis that CCHA1 functions as an ion/H^+^ exchanger. The growth retardation and pale green phenotype of *pam71* mutants could be recovered to the WT level when plants were grown on Mn^2+^-rich medium (Schneider et al., 2016). Moreover, the heterologous expression of PAM71/BICAT1 complemented the Mn^2+^-sensitive phenotype of the *Δpmr1* yeast mutant (Schneider et al., 2016) and increased cytosolic Ca^2+^ concentration in *E. coli* (Frank et al., 2019). Collectively, these results indicate that CCHA1/PAM71/BCAT1 likely transports both Mn^2+^ and Ca^2+^ to control Ca^2+^, Mn^2+^, and H^+^ homeostasis in chloroplasts.

### PGR5 and Other PGR Proteins Are Involved in the Regulation of Luminal pH

PGR proteins were identified through screening of Arabidopsis mutants exhibiting reduced Chl fluorescence quenching (Shikanai et al., 1999). Among them, the well-characterized *pgr1*, *pgr5*, *pgr6*, and *pgr7* mutants exhibited reduced qE (Munekage et al., 2001, 2002; Jung et al., 2010; Martinis et al., 2014). Hence, these PGR proteins directly or indirectly contribute to the regulation of ΔpH and luminal acidification upon light illumination.

PGR5 was first proposed as a component of the ferredoxin (Fd)-dependent CET (the antimycin A-sensitive route), recycling electrons from PSI to the PQ pool (Munekage et al., 2002). Because PGR5 is a small protein without any known motif, the molecular basis of its function is still a topic of debate in the area of photosynthesis-related research. Studies suggest that PGR5 mainly regulates the photosynthetic linear electron transfer (LEF; Tikkanen et al., 2015; Takagi and Miyake, 2018), and the reduction of ΔpH in *pgr5* is caused by the high proton conductivity of thylakoid membranes, possibly *via* the alteration of chloroplast ATP synthase conductivity (*g_H_^+^*; Takagi et al., 2017; Rantala et al., 2020). *g_H_^+^* represents H^+^ permeability across the thylakoid membranes, which is predominantly determined by chloroplast ATP synthase activity (Baker et al., 2007). *g_H_^+^* can be estimated through inverse of the lifetime of the rapid decay signal, upon light-to-dark transitions, of carotenoid absorption changes at 518 or 520 nm in the thylakoid membranes, which is called the electrochromic shift (Baker et al., 2007). By contrast to above arguments about the CET-related function of PGR5, the role of PGR5 in CET is supported by the identification of PGR5-interacting proteins such as PGRL1, Cyt*b_6_f*, and NTRC (DalCorso et al., 2008; Hertle et al., 2013; Nikkanen et al., 2018; Naranjo et al., 2021; Wu et al., 2021). Suppression of LEF in the Δ*5* mutant (Suorsa et al., 2016) and no relationship between elevated *g_H_^+^* and chloroplast ATP synthase activity in the *pgr5* mutant (Yamamoto and Shikanai, 2020) suggest that PGR5 is related to the photosynthetic CET. Nonetheless, the model of PGR5-dependent CET has been challenged by studies on *Chlamydomonas*, which show that PGR5 and PGRL1 indirectly regulate CET (Nawrocki et al., 2019a, 2019b; Buchert et al., 2020).

The *pgr1* mutant harbors a mutation in the *petC* gene, which encodes the Rieske subunit of the Cyt*b_6_f*. In the *pgr1* mutant, Cyt*b_6_f* exhibits a hypersensitive reaction to luminal acidification, which causes abnormal plastoquinol oxidation in and proton translocation through the Cyt*b_6_f* (Munekage et al., 2001; Jahns et al., 2002; Kalituho et al., 2007; Yamamoto and Shikanai, 2019). The *pgr6* mutant harbors a point mutation in the chloroplast *ABC1-like kinase1* (*ABC1K1*) gene and exhibits a disrupted homeostatic relationship between the photoactive PQ pool in thylakoid membranes and the non-photoactive PQ pool in chloroplast plastoglobules, suggesting that PGR6 is involved in PQ homeostasis in chloroplasts (Martinis et al., 2014; Pralon et al., 2019). *PGR7* encodes a chloroplast protein of unknown function, and the *pgr7* mutant is impaired in photosynthetic electron transport (Jung et al., 2010). A variety of PGR proteins and their functions reflect the complexity of the mechanistic regulation of ∆pH across thylakoid membranes and that of *pmf*.

### FLAP1 Is a Novel Regulatory Factor Controlling Chloroplast pH

Fluctuating Light Acclimation Protein1 (FLAP1) was reported as a new NPQ regulatory protein (Sato et al., 2017; Trinh et al., 2019). FLAP1 is evolutionarily conserved among oxygenic phototrophs, exhibits a transmembrane helix with an unknown functional domain (DUF1517), and localizes in both the thylakoid membranes and chloroplast envelope when it is overexpressed (Sato et al., 2017). The Arabidopsis *flap1* mutant exhibits significantly pale green leaves with small chloroplasts under fluctuating light conditions only, indicating that FLAP1 plays a key role in the acclimation to such conditions (Sato et al., 2017). The *flap1* mutant inhibits reduced P700^+^ and qE relaxation upon light–dark transition, indicating that lumen acidification may be maintained at higher levels in the *flap1* mutant than in the WT (Sato et al., 2017). Characterization of *npq4 flap1* and *pgr5 flap1* double mutants revealed that FLAP1 controls PsbS-dependent quenching through the regulation of H^+^ extrusion from the thylakoid lumen to the stroma and possibly from the stroma to the cytosol (Trinh et al., 2019). Indeed, *flap1* mutation partly rescued the lowered induction of steady-state qE in the *pgr5* mutant, suggesting that ∆pH may be maintained at higher levels in the *flap1 pgr5* double mutant than in the *pgr5* single mutant (Trinh et al., 2019). The characterization of FLAP1 homolog A (FlpA) in *Synechocystis* sp. PCC6803 provides further insights into its biological function (Inago et al., 2020). The Δ*FlpA* mutant exhibited retarded growth under fluctuating light conditions and unusual H^+^ extrusion into and H^+^ uptake from the medium upon illumination (Inago et al., 2020). These results indicate that FlpA controls H^+^ translocation across the thylakoid and cytoplasmic membranes to modulate the composition of *pmf*. However, FLAP1- and FlpA-interacting proteins have not yet been identified, which questions the role of FLAP1 in pH homeostasis at the molecular level.

## H^+^-Dependent Transporters and Regulators Control Stromal pH

Genetic approaches identified several H^+^-dependent transporters, exchangers, and regulators, which are localized in the inner envelope membranes and play direct roles in stromal pH regulation (Table 2). Two K^+^ efflux antiporters, KEA1 and KEA2, localized in the inner envelope membranes, are required for osmotic stress responses and chloroplast development (Aranda-Sicilia et al., 2012, 2016; Kunz et al., 2014; Stephan et al., 2016; Tsujii et al., 2019). These antiporters belong to the CPA superfamily (Aranda-Sicilia et al., 2012; Tsujii et al., 2019), which includes Na^+^-H^+^ exchangers (NHXs; CPA1 subfamily), K^+^ efflux antiporters, and cation-H^+^ exchangers (CHXs; CPA2 subfamily; Chanroj et al., 2012). The amino acid sequences of Arabidopsis KEA1 and KEA2 exhibit 77% identity (Aranda-Sicilia et al., 2012). They both possess a chloroplast-targeting signal peptide and a long soluble amino acid chain at the N-terminus, 12 transmembrane helices, and a regulatory K^+^ transport and NAD-binding (KTN) domain at the C-terminus (Aranda-Sicilia et al., 2012; Bölter et al., 2019). Topological analyses of KEA1 suggest that both the long soluble N-terminus and the C-terminal KTN domain lay exposed in the chloroplast stroma but not in the intermembrane space (Bölter et al., 2019). The K^+^ transport activity of KEA1 and KEA2 has been verified through complementation assays in yeast and *E. coli* (Aranda-Sicilia et al., 2012; Tsujii et al., 2019), and their K^+^/H^+^ antiport activity has been confirmed by experiments in intact chloroplasts (Aranda Sicilia et al., 2021). Indeed, the K^+^/H^+^ exchange activity of chloroplasts upon illumination was examined by early studies (Demmig and Gimmler, 1983; Wu et al., 1991; Wu and Berkowitz, 1992). Knocking out either *KEA1* or *KEA2* results in no visible effect on plant growth (Kunz et al., 2014); however, knocking out both genes together reduces plant growth and induces leaf yellowing (Kunz et al., 2014), suggesting that K^+^/H^+^ exchange and pH homeostasis in the stroma are important for chloroplast development and photosynthesis. Recently, the function of KEA1 and KEA2 was shown to be suppressed upon dark-to-light transitions for maintaining alkaline pH levels in the stroma, but their function was fully activated upon light-to-dark transitions to neutralize stromal pH (Aranda Sicilia et al., 2021).

**Table 2:** Proton-involved transporters and protein factors regulate chloroplast stromal pH.

Locus	Protein	Subcellular localization	Proposed function(s)	References
At1g01790	KEA1	Chloroplast envelope membrane	K^+^/H^+^ specific antiporters Regulation of K^+^-induced stromal alkalization upon dark-to-light transition. Regulation of neutralization of stromal pH upon light-to-dark transition.	Aranda-Sicilia et al., 2012 Kunz et al., 2014 Aranda Sicilia et al., 2021
At4g00630	KEA2	Chloroplast envelope membrane (distinct spots)^*^		
At4g13590	PAM71-HL BICAT2	Chloroplast envelope membrane	Putative Ca^2+/^H^+^ antiporter Mn^2+^, Ca^2+^, and pH homeostasis in chloroplasts Bivalent cation transporter	Schneider et al., 2016 Frank et al., 2019
At3g19490	NHD1	Chloroplast envelope membrane	A putative Na^+^/H^+^ antiporter Generation of a sodium gradient across the envelope membrane for activating Na^+^-dependent metabolite transporter Balancing between Na^+^ influxes and effluxes in chloroplasts	Furumoto et al., 2011 Kunz et al., 2014
–	P-type H^+^-ATPase(s)	Chloroplast envelope membrane	Generation of ΔpH across the chloroplast envelope Stabilization of alkaline pH in chloroplast stroma upon light illumination	Berkowitz and Peters, 1993 Shingles and McCarty, 1994 Peters and Berkowitz, 1998
At4g31040	DLDG1	Chloroplast envelope membrane	NPQ control Chloroplast pH homeostasis controller A putative K^+^(Ca^2+^)/H^+^ antiporter or a K^+^(Ca^2+^)/H^+^ antiport regulator	Harada et al., 2019
Atcg00530	Ycf10	Chloroplast envelope inner membrane	Ci transport candidate or regulator of HCO_3_^−^ and CO_2_ uptake^♠^ Regulation of light-induced H^+^ extrusion^♦^ NPQ control^♣^	Rolland et al., 1997 Sasaki et al., 1993 Sonoda et al., 1998 Trinh et al., 2021

* Distinct spots were proposed as the thylakoid biogenesis center.

♠ A study in *Chlamydomonas*.

♦ A study of Ycf10 ortholog (pxcA or CotA) in cyanobacteria.

♣ A study in tobacco.

CHX23, another member of the CPA superfamily, was shown to localize to the chloroplast inner envelope membranes and to function as a putative Na^+^(K^+^)/H^+^ antiporter for adjusting pH in the cytosol while maintaining alkaline pH in the chloroplast stroma (Mäser et al., 2001; Song et al., 2004). The *chx23* mutants were sensitive to salinity stress (Song et al., 2004), suggesting that CHX23 protected plant cells against high cytosolic Na^+^ concentrations. Additionally, CHX23 shows high sequence similarity with NhaS3, a thylakoid membrane-localized Na^+^/H^+^ antiporter in *Synechocystis* (Tsunekawa et al., 2009). Also, the *nhaS3* mutant is sensitive to high salt concentration, indicating that NhaS3 potentially transports Na^+^ from the cytosol to the thylakoid lumen based on light-induced ΔpH across thylakoid membranes (Tsunekawa et al., 2009). By contrast, recent studies about *chx23* mutant and CHX23 functions have been strongly argued such that CHX23 localizes in the endoplasmic reticulum, but not in chloroplasts, and its function is involved in the pollen growth (Lu et al., 2011; Gao et al., 2021).

Another factor that potentially controls H^+^ extrusion across the envelope membrane is H^+^-ATPase, which belongs to the P-type ATPase superfamily (Peters and Berkowitz, 1998). Although genes encoding 11 P-type autoinhibited H^+^-ATPases (AHA1–AHA11) have been identified in Arabidopsis, none of these proteins localize to the chloroplast (Axelsen and Palmgren, 2001; Dall’Osto et al., 2017; Zhang et al. 2017). However, some H^+^-ATPase activity could be detected in inner membranes *in vitro* (Berkowitz and Peters, 1993; Shingles and McCarty, 1994). This suggests that a chloroplast envelope-localized H^+^-ATPase exists and acts as an H^+^ pump. This H^+^-ATPase activity is presumed to be important for the maintenance of light-induced stromal alkalization (Maury et al., 1981; Wu et al., 1991). In fact, Peters and Berkowitz (1998) isolated a chloroplast inner envelope-localized P-ATPase H^+^ pump using radiolabeled [γ-^32^P]ATP (Peters and Berkowitz, 1998). By contrast, proteome analysis of the chloroplast envelope membranes could not identify any P-type H^+^ ATPases, or subunit of V-type and F-type H^+^ ATPases (Ferro et al., 2003; Rolland et al., 2003; Bouchnak et al., 2019). The identified P-type H^+^ ATPase had a molecular weight of 103 kDa, and its dephosphorylation was stimulated by K^+^, which was reached to the highest level at pH 7.5 (Peters and Berkowitz, 1998). Notably, these characteristics were similar to not only those of typical P-type H^+^ ATPases, but also those of P-type ATPases. For example, molecular mass of P-type ATPases vary from 65 to 150 kDa (Dach and Nissen, 2013). Also, K-binding site (Asp617) is conserved among reported P-type ATPases (Buch-Pedersen et al., 2006). Moreover, proteome analysis of the chloroplast envelope membranes detected a heavy metal ATPase1 (HMA1, AT4G37270.1) and a putative HMA6 (AT4G33520.2; Ferro et al., 2003; Rolland et al., 2003; Bouchnak et al., 2019), both of which belong to P-type ATPases. These results suggest that Peters and Berkowitz (1998) investigated HMA1 or HMA6, but not a P-type H^+^ ATPase.

A novel NPQ regulatory protein, Day-Length-dependent Delayed-Greening1 (DLDG1), has been proposed to contribute to H^+^ extrusion across the envelope membranes in chloroplasts (Harada et al., 2019). The envelope membrane-localizing mature DLDG1 protein contains an extended N-terminus, three transmembrane helixes, and a conserved motif at the C-terminus (Harada et al., 2019). Interestingly, the nuclear gene-encoded DLDG1 protein exhibits amino acid sequence similarity with the plastid gene-encoded Ycf10, which also localizes to the chloroplast envelope membranes (Harada et al., 2019). Heterologous expression of *DLDG1* and *Ycf10* in *E. coli* K^+^ uptake- and Na^+^ antiporter-deficient strains indicates that DLDG1 could complement the transport deficiency of both K^+^ and Na^+^, whereas Ycf10 is able to complement that of Na^+^ alone (Harada et al., 2019). This finding suggests that DLDG1 and Ycf10 are involved in Na^+^/H^+^ and/or K^+^/H^+^ antiport in *E. coli*. The Arabidopsis *dldg1* mutant exhibits pale green young leaves and abnormal chloroplast structures, indicating that DLDG1 is important for chloroplast development during the early stages of leaf development, possibly because of its influence on ion/H^+^ homeostasis in the stroma (Harada et al., 2019). Strikingly, a sustained induction and slow relaxation of qE were observed in the *dldg1* mutant, likely because of strong luminal acidification upon illumination (Harada et al., 2019). In addition, both the PSII quantum yield (Y[II]) and PSI donor-side limitation (Y[ND], a non-photochemical quantum yield measure) were reduced in the *dldg1* mutant compared with the WT, suggesting that luminal pH was lower in the mutant than in the WT, thus inhibiting the transfer of electrons from PSII to PSI (Harada et al., 2019). Although the molecular mechanisms underlying the regulatory function of DLDG1 remain unclear, researchers suggested that DLDG1 regulates chloroplast H^+^ homeostasis through H^+^ extrusion from the stroma to the cytosol (Harada et al., 2019). Moreover, studies on DLDG1 homologs (PxcA and PxcL) in cyanobacteria show that these homologs control H^+^ extrusion and uptake across plasma membranes (Katoh et al., 1996; Sonoda et al., 1998; Inago et al., 2020).

Studies on the DLDG1 homolog, Ycf10, in pea (CemA; Sasaki et al., 1993) and *Chlamydomonas* (Rolland et al., 1997) suggest the involvement of Ycf10 in plastid pH regulation and redox balance in chloroplast envelope (Jäger-Vottero et al., 1997; Rolland et al., 1997). Researchers hypothesized that H^+^ extrusion regulated by Ycf10/CemA is important in the acidification of intermembrane spaces for the conversion of HCO_3_^−^ to CO_2_, which in turn accelerates the diffusion of CO_2_ into the chloroplasts (Rolland et al., 1997). Tobacco *ycf10* loss-of-function mutants also showed excessive induction of NPQ, similar to that observed in Arabidopsis *dldg1* mutants (Trinh et al., 2021). However, *g_H_^+^* increased in *ycf10* mutants but decreased in *dldg1* mutants (Harada et al., 2019; Trinh et al., 2021). Furthermore, NPQ decreases in *ycf10* and increases in *dldg1* mutants with the increase in the duration of fluctuating light conditions (Harada et al., 2019; Trinh et al., 2021). Collectively, these results suggest that DLDG1 and Ycf10 distinctively control H^+^ extrusion in chloroplasts (Trinh et al., 2021). Complementation assays of *E. coli* antiporter mutants suggest functional interaction between DLDG1 and Ycf10 for controlling Na^+^ extrusion and K^+^ uptake (Trinh et al., 2021).

## Regulation of pH Homeostasis in Chloroplasts

As mentioned above, pH in all chloroplast compartments is stably maintained at neutral levels in the dark. However, upon dark-to-light transitions, pH increases to alkaline levels in chloroplast stroma and decreases to acidic values in the thylakoid lumen. During light periods, stromal pH is constantly maintained at alkaline levels, whereas thylakoid luminal pH is stabilized at acidic levels. The distinct pH levels in chloroplast stroma and thylakoid lumen return to neutral levels upon light-to-dark transitions (Heldt et al., 1973). As pH in chloroplast compartments is stably maintained at appropriate levels during dark and light periods, the regulation of H^+^ transport across chloroplast membranes is essential for maintaining chloroplast pH homeostasis (Höhner et al., 2016). It is noteworthy that stable pH levels in chloroplasts are also caused by the buffering ability of chloroplast metabolites (Wegner and Shabala, 2020).

**Figure 3:**
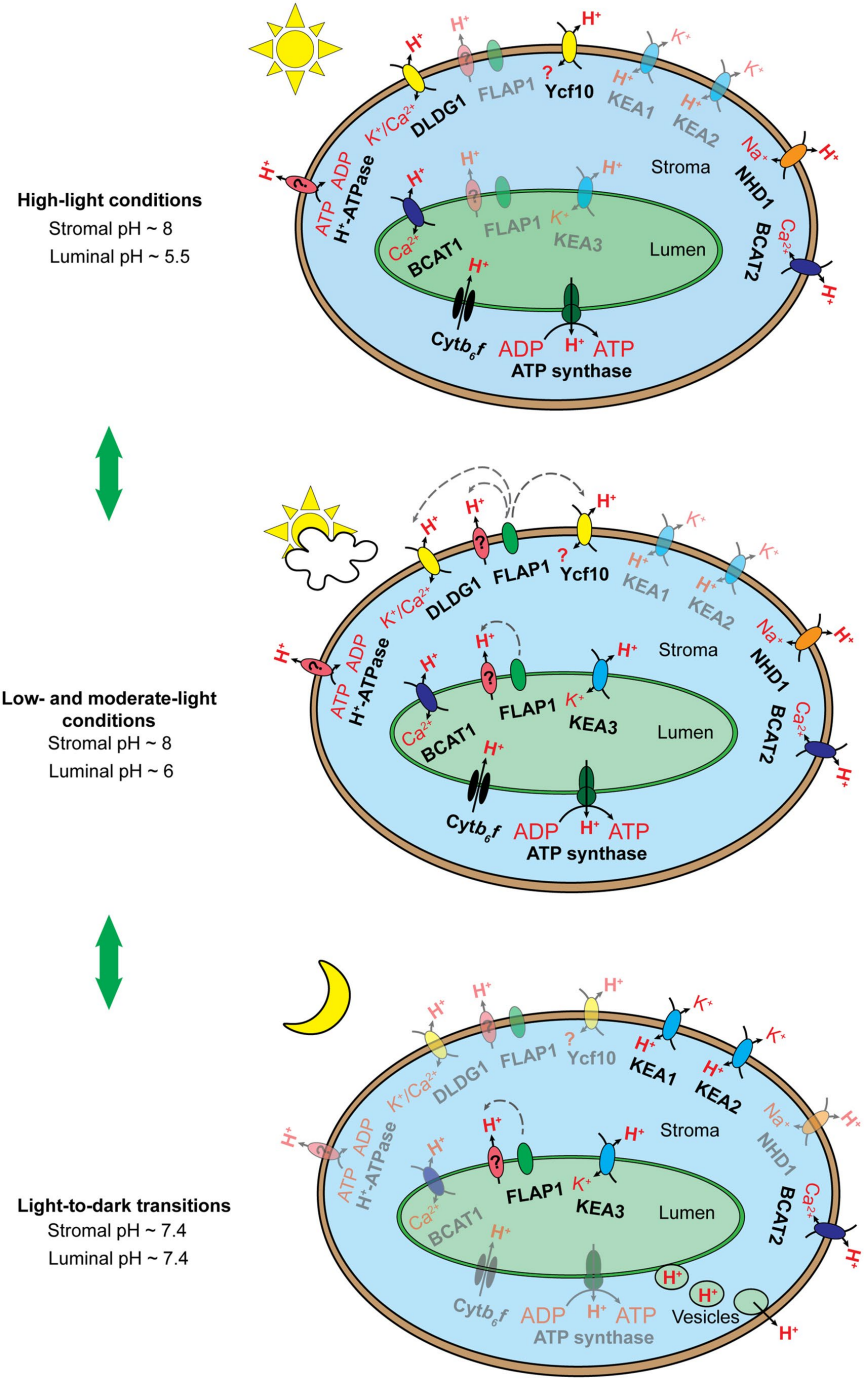
Schematic illustration of the hypothesized chloroplast pH homeostasis *via* H^+^ transport pathway involving chloroplast membrane-localized transporters, exchangers, and protein complexes under high light conditions (top), low and moderate light conditions (middle), and upon light-to-dark transitions (bottom). The number of proteins shown does not reflect the molecular stoichiometry between them. Black arrows indicate ion/H^+^ flow. Gray dashed arrows indicate the inductive effect of a regulator toward its target transporters. Fully active transporters, exchangers, and protein complexes are 100% opaque, whereas their less active or inactive counterparts are shown with ~50% opacity. The Cyt*b_6_f* complex and chloroplast ATP synthase are activated by light-induced electron transfer chains and the *pmf*, respectively, under light conditions (top, middle) and are deactivated upon light-to-dark transitions (bottom). KEA3 performs little function under high light conditions (top) but more function under low and moderate light conditions (middle), as discussed by Armbruster et al. (2016). We proposed that KEA3 also acts to neutralize chloroplast pH upon the light-to-dark transition (bottom). FLAP1 potentially regulates an unknown H^+^ transporter and/or exhibits functional interaction with DLDG1 and Ycf10, as suggested previously (Harada et al., 2019; Inago et al., 2020). FLAP1 relaxes NPQ induction and shows higher activity under low and moderate light conditions (middle) than under high light conditions (bottom), as discussed previously (Sato et al., 2017; Trinh et al., 2019). We proposed that FLAP1 also contributes to the neutralization of chloroplast pH upon light-to-dark transitions (bottom). BICAT1 uptakes Ca^2+^ under light conditions (Frank et al., 2019). The *bicat1* mutants showed lower NPQ induction (Wang et al., 2016; Frank et al., 2019), although BICAT1 is proposed to transport H^+^ from the thylakoid lumen to the chloroplast stroma. This can be explained by the loss of OEC in the *bicat1* mutants, because of the reduction in the Mn^2+^ content of chloroplasts, which suppresses either light-induced electron transfer chains or H^+^ translocation across thylakoid membranes (Schneider et al., 2016). Next, BICAT1 functions under light conditions (top, middle), as shown by Frank et al. (2019). As Ca^2+^ accumulation in the chloroplast stroma contributes to the downregulation of CO_2_ fixation, because of the inhibition of several enzymes involved in the CBB cycle (Rocha and Vothknecht, 2012), and to the transcription of plastidial genes *via* the synthesis of the secondary messenger, guanosine tetraphosphate (Ono et al., 2019), BICAT1 is proposed to be deactivated upon the light-to-dark transition (bottom). Both KEA1 and KEA2 antiporters are less active under light conditions (top, middle) than upon light-to-dark transitions (bottom), as reported previously (Aranda Sicilia et al., 2021). The BICAT2 antiporter is active under light conditions (top, middle), as reported by Frank et al. (2019). As mentioned above, Ca^2+^ uptake by the chloroplast stroma is essential for the suppression of CO_2_ fixation under stress conditions and upon light-to-dark transitions. Thus, we proposed that BICAT2 is activated upon light-to-dark transitions (bottom). DLDG1, Ycf10, NHD1, and H^+^-ATPase are all proposed to contribute to H^+^ extrusion into the cytosol, thus contributing to the maintenance of alkaline pH in the chloroplast stroma under light conditions (top, middle). The functions of proteins are deactivated upon light-to-dark transitions (bottom). Direct H^+^ export from the thylakoid lumen to the cytosol is proposed to occur upon light-to-dark transitions (bottom) to neutralize the luminal pH. This mechanism might occur at contact sites between thylakoid and envelope membranes (data not shown) or through vesicle transport from thylakoids to chloroplast envelope membranes (bottom).

Under light conditions, the maintenance of proper alkaline pH in the chloroplast stroma is controlled through two primary regulatory mechanisms, which counteract passive H^+^ diffusion from the cytosol to the chloroplast stroma (Höhner et al., 2016): (i) light-dependent H^+^ flux into the thylakoid lumen and (ii) H^+^ extrusion across the envelope membranes (Figure 3). The former mechanism is mainly involved in Cyt*b_6_f* activity, while the latter is regulated by H^+^-related transporters localized in the chloroplast inner envelope membranes. Notably, the latter mechanism is challenged by Elsässer et al. (2020). In addition, the theory about light-induced electron transport coupled with H^+^ translocation across the chloroplast envelope membrane is plausible, since the components of an electron transfer chain have been identified in chloroplast envelope membranes (Jäger-Vottero et al., 1997; Murata and Takahashi, 1999); however, chloroplast envelope-localized proteins that function like Cyt*b_6_f* have never been identified (Jäger-Vottero et al., 1997; Höhner et al., 2016). In the thylakoid lumen, pH is stabilized at acidic levels by balancing H^+^ influx into the thylakoid lumen with H^+^ efflux into the chloroplast stroma. Regulation of H^+^ influx mainly involves the activity of OEC at PSII and the Q cycle in the Cyt*b_6_f*, whereas the H^+^ efflux is controlled by ion/H^+^ antiporters (e.g., KEA3) and chloroplast ATP synthase.

Upon the light-to-dark transition, pH in the chloroplast stroma decreases from alkaline to neutral levels because of H^+^ efflux from the thylakoid lumen (Heldt et al., 1973) and H^+^ uptake *via* chloroplast envelope membrane-localized ion/H^+^ antiporters (Figure 3; Aranda Sicilia et al., 2021). Acidic pH levels in the thylakoid lumen are also neutralized by H^+^ efflux into the chloroplast stroma and direct H^+^ export from the thylakoid lumen to the cytosol (Figure 3; Aranda Sicilia et al., 2021). H^+^ efflux from the thylakoid lumen to the chloroplast stroma may be controlled by the thylakoid membrane-localizing ion/H^+^ antiporters. After turning off moderate actinic light, the *kea3* mutants exhibit slower relaxation of NPQ in comparison with the WT (Wang et al., 2017; Wang and Shikanai, 2019), suggesting that KEA3 contributes to H^+^ efflux into the chloroplast stroma. Moreover, H^+^ efflux from the thylakoid lumen into the chloroplast stroma is not sufficient for neutralizing pH both in the stroma and lumen upon light-to-dark transitions (Aranda Sicilia et al., 2021). Therefore, H^+^ uptake *via* chloroplast envelope membranes and direct H^+^ export from the thylakoid lumen to the cytosol were proposed as additional mechanisms. The former mechanism was verified by functional characterization of KEA1 and KEA2 (Aranda Sicilia et al., 2021); however, still, no direct evidence exists supporting the later mechanism previously hypothesized (Heber and Heldt, 1981). In fact, the formation of chloroplast vesicles and direct contact sites between thylakoid and envelope membranes has been recently reported (Vothknecht and Westhoff, 2001; Westphal et al., 2003; Vothknecht et al., 2012; Khan et al., 2013; Rast et al., 2015; Lindquist and Aronsson, 2018). Because of its dual localization to both the thylakoid and envelope membranes, FLAP1 demonstrates potential to be involved in the direct H^+^ export from the thylakoid lumen to the cytosol. Both DLDG1 and Ycf10 localize to the envelope membranes, yet they control luminal pH, suggesting that these proteins might be involved in the proposed mechanism as well.

## Identification of New Factors Controlling Chloroplast pH Homeostasis

Knowledge gap in chloroplast pH homeostasis demands the identification of novel proteins and of functional interaction between the identified proteins. Understanding how many genes are required for the proper function of chloroplasts is important. Based on the endosymbiosis theory, the chloroplast originated from an ancient cyanobacterium (Gould et al., 2008). Since then, a large number of endosymbiont genes have been transferred to the host nuclear genome. The cyanobacterium *Anabaena* sp. PCC7120 exhibits 5,366 genes, whereas plastid genomes of the red alga *Porphyra purpurea* and the parasitic plant *Epifagus virginiana* possess only 251 and 42 genes, respectively (Gould et al., 2008). In Arabidopsis, ~3,000 nuclear genes encode plastid−/chloroplast-localized proteins (The Arabidopsis Genome Initiative, 2000; Savage et al., 2013), while the plastid genome contains only 87 protein-coding genes (Sato et al., 1999). In other words, nearly 97% of plastid proteins are synthesized outside chloroplasts and then imported into the chloroplasts (Cline and Dabney-Smith, 2008). Proteomic analysis of Arabidopsis identified 1,323 chloroplast-localized proteins, of which 819 precisely showed sub-plastidial localization (Ferro et al., 2010; Bruley et al., 2012). Proteomic analysis of purified chloroplast envelope membranes revealed 462 envelope-associated proteins per 1,269 identified proteins (Bouchnak et al., 2019). Based on sequence similarity, 100 and ~ 90 envelope proteins were identified as metabolic factors and transporters, respectively, though 16% (~70 proteins) over 462 envelope proteins are still unknown (Bouchnak et al., 2019).

Forward and reverse genetics approaches serve as powerful tools for the identification and characterization of the biological functions of unknown proteins. Indeed, forward genetic screening of mutagenized plant libraries identified many important photosynthetic regulatory proteins such as PGRs, NPQs, high chlorophyll fluorescence (HCFs), and Hunger for Oxygen in Photosynthetic Electron transport reaction (HOPEs; Meurer et al., 1996; Niyogi et al., 1998; Shikanai et al., 1999; Meierhoff et al., 2012; Dall’Osto et al., 2017; Takagi et al., 2017). Most proteins identified by the forward genetics are involved in controlling light-induced electron transport and H^+^ translocation across the thylakoid membranes. However, it seems likely that high chlorophyll fluorescence is not a good phenotype for selecting mutants impaired in pH homeostasis in the chloroplast stroma, since the alteration of protein levels itself significantly affects the quantum yield of fluorescence.

Reverse genetics also led to the identification of many important proteins involved in H^+^ transport across thylakoid and envelop membranes in chloroplasts. All identified transporters share high evolutionary conservation, belong to a specific transporter family, and contain the chloroplast signal peptide. For example, KEA1, KEA2, and KEA3 are homologs of bacterial KefC, which belongs to the CPA superfamily, and contain chloroplast transit peptides (Aranda-Sicilia et al., 2012, 2016; Kunz et al., 2014). In addition, DLDG1, Ycf10, and FLAP1 were identified through reverse genetic screening based on the following properties: (i) predicted as chloroplast proteins; (ii) co-expression with known NPQ-related genes; and (iii) high sequence conservation among oxygenic phototrophs (Sato et al., 2017; Harada et al., 2019). Similarly, gene co-expression databases (e.g., ATTED-II) or protein–protein association networks (e.g., STRING) could be used to identify novel pH homeostasis-related proteins that co-express or/and are associated with known proteins. Such screening may further identify new players involved in the regulation of pH homeostasis in chloroplasts.

## Concluding Remarks

This review summarizes our current understanding of pH homeostasis in chloroplasts and its role in photosynthetic regulation. Although the importance of chloroplast pH homeostasis and the role of pH as a signal/messenger were proposed a long time ago, the unknown identity of proteins involved in the proposed mechanisms has been creating many obstacles in fully understanding the significance of pH homeostasis in chloroplasts. Further research is needed to identify novel chloroplast homeostasis-related proteins and their interacting partners.

## Author Contributions

MDLT wrote the first draft version of the manuscript. All authors contributed to writing the manuscript. All authors contributed to the article and approved the submitted version.

## Funding

The study was supported by JSPS KAKENHI grant number 22 K06276.

## Conflict of Interest

The authors declare that the research was conducted in the absence of any commercial or financial relationships that could be construed as a potential conflict of interest.

## Publisher’s Note

All claims expressed in this article are solely those of the authors and do not necessarily represent those of their affiliated organizations, or those of the publisher, the editors and the reviewers. Any product that may be evaluated in this article, or claim that may be made by its manufacturer, is not guaranteed or endorsed by the publisher.
